# Editorial: The Cerebellum: From Vascular Disease to Neurodegeneration

**DOI:** 10.3389/fneur.2021.657376

**Published:** 2021-02-23

**Authors:** Sirio Cocozza

**Affiliations:** Department of Advanced Biomedical Sciences, University of Naples “Federico II”, Naples, Italy

**Keywords:** cerebellum, cognition, magnetic resonance imaging, neurodegenaration, neuroinflammation

The cerebellum (from the Latin “little brain”), despite representing only 10% of the total brain volume, contains more than 50% of the total number of neurons of the central nervous system (CNS). The abundance of neuronal elements is only one of the many intriguing features of this structure, the exact role of which is still highly debated. Indeed, the number of studies dedicated to the investigation of the cerebellum has significantly increased over the last few years (1). Nevertheless, although many data have been gathered and many theories have been proposed, we are still far from understanding the exact function of this structure. The classical theories focused on the role of the cerebellum in fine-tuning of muscle control have been widely reconsidered, and new hypotheses have been advanced (2). These include its role as a sensory acquisition device, beyond motor control and learning, as well as a pivotal role in cognition, with a recognized cerebellar participation in a variety of cognitive functions (ranging from mood control to language, memory, attention, and spatial data management) (3–5), and pathological conditions (6–13).

This Research Topic gathers 5 studies all conducted with the aim of expanding our knowledge about cerebellar pathophysiology. This research covered different aspects of cerebellar involvement, from molecular patterns to innovative tools for clinical evaluation of patients with cerebellar symptoms, as well as a relatively wide spectrum of *in-vivo* application of advanced imaging techniques.

In the first study of this Research Topic, Lin et al. reviewed the current knowledge about the potential relationship between molecular patters, functional topography images and clinical cerebellar disorders. In particular, they reviewed the relation between the pattern of zebrin distribution, which is a glycolytic enzyme expressed in cerebellar Purkinje cells, and cerebro-cerebellar connections. Knowing the distribution of zebrin-negative and zebrin-positive zones, receiving respective inputs from somatosensory and non-motor regions, as well as the role of cerebellar damage shown by structural ad functional MRI studies in different neurodegenerative disorders, the Authors hypothesized that zebrin-positive cells could contribute to the compensatory phenomena observed in conditions showing cerebellar-related damage.

The second study of this Research Topic, presented by Honda et al. proposed an innovative method to quantitatively evaluate clinical damage in conditions presenting with cerebellar involvement. Indeed, the Authors developed a novel Kinect sensor system to obtain quantitative and reliable measurements of functions usually explored in patients with cerebellar symptoms (namely, gait and upper limb coordination). Interestingly, this examiner-independent device not only proved to evaluate movements more accurately than with the naked eye but also provided additional information about the ataxic movements usually not obtained through the standard clinical evaluation.

The remaining 3 studies included in this Research Topic applied advanced imaging techniques to evaluate *in-vivo* the degree of cerebellar involvement in conditions characterized by a predominant involvement of this structure (such as hereditary degenerative ataxias), or widespread CNS disorders in which cerebellar damage appears to be a significant, although relatively understudied, feature (namely, Multiple Sclerosis-MS).

Bando et al. investigated cerebellar gray matter volume changes in Spinocerebellar Ataxia (SCA) patients via a voxel-based morphometry (VBM) analysis, to evaluate the relationship between regional patterns of cerebellar volume loss and adaptive learning abilities. Compared to healthy controls, SCA patients showed reduced gray matter volume affecting both cerebellar hemispheres, and in particular areas including lobules IV-VIII, with the adaptive learning index correlating with the degree of cerebellar volume loss. The results of this study further indicate the crucial contribution of cerebellar cortex to sensory-motor functions, with the Authors speculating about a possible application of the identified changes as reliable biomarkers of disease severity and progression in SCA patients.

The study by Park et al. investigated a different feature of cerebellar involvement in SCA (namely, the microstructural white matter integrity), via a diffusion MRI (dMRI) analysis. The Authors presented cross-sectional and longitudinal dMRI data in SCA patients, applying multiple analysis approaches to provide complementary information about white matter microstructural abnormalities in these conditions. At the cross-sectional analyses they found relatively consistent alterations within several white matter tracts across different analysis methods, although certain differences were identified only using specific approaches, thus demonstrating the unique contribution of each pipeline in providing a comprehensive understanding of microstructural changes in these patients. Furthermore, their longitudinal analysis showed that the middle cerebellar peduncles could be specifically affected in SCA1 patients, with microstructural alterations being already present in the pre-clinical stage, therefore suggesting that this region might represent a good candidate biomarker to monitor disease progression.

Finally, the work by Ruggieri et al. investigated the presence of cerebellar damage in MS patients by evaluating the relation occurring between regional cerebellar cortical atrophy, microstructural damage of the cerebellar peduncles, and physical disability. By integrating different imaging techniques, the Authors were able to confirm the involvement of the predominantly sensorimotor areas of the cerebellar cortex, and show how damage of these regions was able to explain part of the physical deficits occurring in MS patients. Furthermore, they proved how microstructural alterations of both afferent and efferent cerebellar connections were correlated to cerebellar lobular atrophy in this condition, lending further evidence to the hypothesis of an impaired input integration as one of the major causes of physical disability in MS.

All the Articles published within this Research Topic provide additional and significant contributions to our understanding of how the cerebellum participates in all the different aspects of motor and non-motor behavior. In particular, these Articles further support the central role of advanced MRI techniques in providing a non-invasive evaluation of macro- and microstructural integrity in different neurodegenerative and neuroinflammatory conditions affecting the CNS, further contributing to the complex task of mapping cerebellar functions.

## Author Contributions

SC: wrote the manuscript.

## Conflict of Interest

The author declares that the research was conducted in the absence of any commercial or financial relationships that could be construed as a potential conflict of interest.
